# Mesenchymal stromal cell therapy attenuated lung and kidney injury but not brain damage in experimental cerebral malaria

**DOI:** 10.1186/s13287-015-0093-2

**Published:** 2015-05-22

**Authors:** Mariana C Souza, Johnatas D Silva, Tatiana A Pádua, Natália D Torres, Mariana A Antunes, Debora G Xisto, Thiago P Abreu, Vera L Capelozzi, Marcelo M Morales, Ana A. Sá Pinheiro, Celso Caruso-Neves, Maria G Henriques, Patricia RM Rocco

**Affiliations:** Laboratory of Applied Pharmacology, Farmanguinhos, Oswaldo Cruz Foundation, Av Brasil, 4365, Manguinhos, CEP–21040-900 Rio de Janeiro, RJ Brazil; Laboratory of Pulmonary Investigation, Carlos Chagas Filho Institute of Biophysics, Federal University of Rio de Janeiro, Av Carlos Chagas Filho, 373 Bloco G, Cidade Universitária, CEP–21941-902 Rio de Janeiro, RJ Brazil; Laboratory of Biochemistry and Cellular Signaling, Carlos Chagas Filho Institute of Biophysics, Federal University of Rio de Janeiro, Av Carlos Chagas Filho, 373 Bloco G, Cidade Universitária, CEP–21941-902 Rio de Janeiro, RJ Brazil; Department of Pathology, Faculty of Medicine, University of São Paulo, Av. Dr. Arnaldo, 455, Cerqueira César, CEP–01246903 São Paulo, SP Brazil; Laboratory of Cellular and Molecular Physiology, Carlos Chagas Filho Institute of Biophysics, Federal University of Rio de Janeiro, Av Carlos Chagas Filho, 373 Bloco G, Cidade Universitária, CEP–21941-902 Rio de Janeiro, RJ Brazil; National Institute for Science and Technology on Innovation on Neglected Diseases (INCT/IDN), Center for Technological Development in Health (CDTS), Oswaldo Cruz Foundation (Fiocruz), Av Brasil, 4365, Manguinhos, CEP–21040-900 Rio de Janeiro, RJ Brazil

## Abstract

**Introduction:**

Malaria is the most relevant parasitic disease worldwide, and still accounts for 1 million deaths each year. Since current antimalarial drugs are unable to prevent death in severe cases, new therapeutic strategies have been developed. Mesenchymal stromal cells (MSC) confer host resistance against malaria; however, thus far, no study has evaluated the therapeutic effects of MSC therapy on brain and distal organ damage in experimental cerebral malaria.

**Methods:**

Forty C57BL/6 mice were injected intraperitoneally with 5 × 10^6^*Plasmodium berghei*-infected erythrocytes or saline. After 24 h, mice received saline or bone marrow (BM)-derived MSC (1x10^5^) intravenously and were housed individually in metabolic cages. After 4 days, lung and kidney morphofunction; cerebrum, spleen, and liver histology; and markers associated with inflammation, fibrogenesis, and epithelial and endothelial cell damage in lung tissue were analyzed.

**Results:**

In *P. berghei*-infected mice, BM-MSCs: 1) reduced parasitemia and mortality; 2) increased phagocytic neutrophil content in brain, even though BM-MSCs did not affect the inflammatory process; 3) decreased malaria pigment detection in spleen, liver, and kidney; 4) reduced hepatocyte derangement, with an increased number of Kupffer cells; 5) decreased kidney damage, without effecting significant changes in serum creatinine levels or urinary flow; and 6) reduced neutrophil infiltration, interstitial edema, number of myofibroblasts within interstitial tissue, and collagen deposition in lungs, resulting in decreased lung static elastance. These morphological and functional changes were not associated with changes in levels of tumor necrosis factor-α, keratinocyte-derived chemokine (KC, a mouse analog of interleukin-8), or interferon-γ, which remained increased and similar to those of *P. berghei* animals treated with saline. BM-MSCs increased hepatocyte growth factor but decreased VEGF in the *P. berghei* group.

**Conclusions:**

BM-MSC treatment increased survival and reduced parasitemia and malaria pigment accumulation in spleen, liver, kidney, and lung, but not in brain. The two main organs associated with worse prognosis in malaria, lung and kidney, sustained less histological damage after BM-MSC therapy, with a more pronounced improvement in lung function.

## Introduction

Malaria is the most relevant parasitic disease worldwide. Despite efforts toward its eradication, malaria still accounts for 1 million deaths each year [1]. Cerebral malaria is characterized by multiple organ dysfunction triggered by circulating parasitized red blood cells (RBCs). Besides the brain, highly vascularized organs such as the lungs and kidneys are especially affected during cerebral malaria. In fact, of patients with cerebral malaria 20–30 % develop acute respiratory distress syndrome (ARDS) [2] and 40–50 % develop acute renal failure [3]. Disruption of the blood–brain barrier (BBB), sequestration of parasitized RBCs in the brain, lung, and kidneys, and a systemic inflammatory response, including production of cytokines and activation of inflammatory cells, have been consistently observed in both human and nonprimate models of cerebral malaria [4]. Recent studies report that current antimalarial drugs are insufficient to prevent death in severe cases of malaria; thus, adjunctive therapies aiming to modulate the systemic inflammatory response triggered by malaria have been proposed [5].

The beneficial effects of cell therapy have been demonstrated not only in infectious diseases [6–8] but also in parasitic diseases [9–12]. Mesenchymal stromal cells (MSCs) attenuated liver injury by diminishing the production of proinflammatory mediators in schistosomiasis [10] and decreased liver fibrosis induced by *Trypanosoma cruzi* infection [11]. Using a model of noncerebral malaria, Belyaev et al. [12] reported that treatment of mice infected with *Plasmodium chabaudi* (a *Plasmodium* species that does not induce cerebral malaria) with lymphoid-primed multipotent progenitor cells decreased parasitemia, probably by inducing a phagocytic active cell population. Accordingly, experimental cerebral malaria (ECM)-resistant mice treated with cells expressing stem cell antigen-1 exhibited decreased parasitemia and an increased survival rate when compared with nontreated mice [13]. However, no study has thus far evaluated the effects of mesenchymal stem cell therapy on brain, spleen, liver, kidney, and lung damage in ECM. In the present study, we hypothesized that bone marrow-derived mesenchymal stromal cells (BM-MSCs) might reduce mortality in ECM by acting not only in the brain but also in other organs.

## Methods

This work was carried out in strict accordance with the recommendations of the US National Research Council *Guide for the Care and Use of Laboratory Animals*. The study protocol was approved by the Committee on Ethical Use of Laboratory Animals of the Oswaldo Cruz Foundation (permit number LW52/12) and by the Research Ethics Committee of the Federal University of Rio de Janeiro Health Sciences Center (CEUA-CCS-019). All animals received humane care in compliance with the “Principles of Laboratory Animal Care” formulated by the National Society for Medical Research.

### Extraction, isolation, and characterization of BM-MSCs

Bone marrow cells were obtained from femurs and tibias. After isolation, 1 × 10^7^ bone marrow-derived cells were cultured (37 °C, 5 % CO_2_) in T25 culture flasks (TPP, Schaffhausen, Switzerland) with Dulbecco’s modified Eagle’s medium (DMEM; Invitrogen, Carlsbad, CA, USA) containing 15 mM HEPES (Sigma, St. Louis, MO, USA), 15 % inactivated fetal bovine serum (FBS; Invitrogen), 100 units/ml penicillin, and 100 mg/ml streptomycin antibiotic solution (Gibco, Carlsbad, MO, USA) [14]. On day 3 of culture, the medium was changed and nonadherent cells were removed. Adherent cells reaching 80 % confluence were passaged with 0.05 % trypsin–Ethylenediaminetetraacetic acid solution (Gibco) and then maintained in DMEM with 10 % FBS (complete medium). At the third passage, approximately 1 × 10^6^ cells were characterized as BM-MSCs according to the International Society of Cellular Therapy Consensus, i.e., adherent to plastic under standard conditions, expressing some surface markers (CD73, CD90, and CD105) and lacking expression of others (CD34, CD45, CD11b, and CD19), and demonstrating capacity to differentiate into mesenchymal lineages under in vitro conditions [15]. Flow cytometry was performed with antibodies against CD45 (leukocytes), CD34 (hematopoietic precursors), CD29 and CD45 (nonhematopoietic precursors), and Sca-1 (stem/progenitor cells) (BD Biosciences, San Jose, CA, USA). The absence of CD34 and CD45 and the presence of CD29 and Sca-1 were used to identify MSCs [16]. To measure the small-angle forward scatter (FSC) intensity (~0–5°) and the limited-angle side scatter (SSC) intensity (~85–95°), a photodiode and a photomultiplier tube were used respectively. Additionally, the potential of MSCs to differentiate into mesenchymal lineages including osteoblasts and chondroblasts under *in vitro* conditions was evaluated. Osteogenic differentiation was induced by culturing MSCs for up to 3 weeks in DMEM 10 % FBS and 15 mM HEPES (Sigma), supplemented with 10^–8^ M/l dexamethasone (Sigma), 5 μg/ml ascorbic acid 2-phosphate (Sigma), and 10 mM/l β-glycerolphosphate (Sigma). To observe calcium deposition, cultures were stained with Alizarin Red S (Nuclear, São Paulo, SP, Brazil). To induce chondrogenic differentiation, MSCs were cultured in DMEM supplemented with 10 ng/ml transforming growth factor (TGF)-β1 (Sigma), 50 nM ascorbic acid 2-phosphate (Sigma), and 6.25 mg/ml insulin for 3 weeks. To confirm differentiation, cells were fixed with 4 % paraformaldehyde in phosphate-buffered saline (PBS) for 1 hour at room temperature and stained with Alcian Blue pH 2.5.

### Animal preparation and experimental protocols

A total of 92 C57BL/6 male mice (6–7 weeks old) were used. In 72 mice, the lung mechanics, renal function, and brain, spleen, liver, kidney, and lung histology were analyzed, and enzyme-linked immunosorbent assay (ELISA) was performed in lung tissue. All experimental conditions were repeated in triplicate (*n* = 6/group). The remaining 20 mice were used to evaluate the survival rate. Mice were provided by the Oswaldo Cruz Foundation breeding unit (Rio de Janeiro, RJ, Brazil) and kept in cages in a room at the Farmanguinhos experimental facility, with free access to food and fresh water, temperature ranging from 22 to 24 °C, and a standard 12-hour light/dark cycle, until experimental use. All animals were randomly assigned to two groups: uninfected or *Plasmodium berghei-*infected. *P. berghei* ANKA GFPcon 259 cl2 was kindly provided by Dr. L. Carvalho (Fiocruz, Rio de Janeiro, RJ, Brazil) and is a donation from the Malaria Research and Reference Reagent Resource Center—MR4 (Manassas, VA, USA; deposited by C.J. Janse and A.P. Waters; MR4 number: MRA-865). Mice were infected by injection intraperitoneally (i.p.) of *P. berghei*-infected RBCs withdrawn from a previously infected mouse (5 × 10^6^ infected RBCs diluted in 200 μl sterile saline solution). Uninfected mice received saline alone (200 μl, i.p.). Twenty-four hours after infection, the uninfected and *P. berghei* groups were further randomized into subgroups to receive saline (0.05 ml) or BM-MSC (1 × 10^5^ in 0.05 ml saline) intravenously into the internal jugular vein. Five days after infection, surviving mice were euthanized by injection i.p. of a mixture of ketamine (100 mg/kg) and xylazine (10 mg/kg) followed by pentobarbital sodium (150 mg/kg). Five days after infection, a thick blood smear was performed for determination of parasitemia by rapid panoptic staining (Laborclin, Paraná, Brazil).

To calculate the survival rate, lethality in the treated (*n* = 10) and untreated (*n* = 10) subgroups of *P. berghei*-infected mice was recorded daily until day 20 post infection.

### Immunofluorescent staining and flow cytometric analysis

Splenocytes from C57BL/6 mice were isolated by Histopaque-1077 (Sigma, St. Loius, MO, USA). One hour after treatment, cells were washed and then incubated in PBS plus 10 % FBS and 0.1 % sodium azide (PBS-S; Sigma-Aldrich) and blocked with FcγIIR monoclonal antibodies (mAbs; 1:100, CD16/CD32; BD Pharmingen, San Jose, CA, USA) for 30 minutes at 4 °C. After blocking, cells were labeled with fluorescein isothiocyanate (FITC)-conjugated mAb anti-mouse CD11b antibodies diluted in PBS-S and incubated for another 30 minutes at 4 °C. Cells were then washed and resuspended in PBS/0.1 % sodium azide for data acquisition in an Accuri flow cytometer (BD Biosciences). FSC and SSC were set to exclude dead cells, and at least 10^4^ lymphocytes were analyzed per sample. Control staining to determine the positive population was performed based on an irrelevant IgG isotype labeled with FITC. Once determined, the gate was rigorously maintained for all analyses. Data analysis was performed using FlowJo software (Tree Star, Inc., Ashland, OR, USA).

### Measurement of renal and lung function

Immediately after treatment with BM-MSCs, mice were allocated individually to metabolic cages and kept in a temperature-controlled room under a 12-hour light/dark cycle, with free access to food and water. After 24 hours of adaptation, 24-hour urine samples were collected from the different experimental groups 1 day before euthanasia, which was performed on postinfection day 5. Urine samples were clarified by centrifugation at 600 × *g* for 5 minutes and the supernatant was separated and stored at −20 °C until use. Urine samples were assayed colorimetrically to determine total protein levels, using specific commercially available kits (Gold Analisa kit 498 M; Gold Analisa Diagnóstica, Belo Horizonte, MG, Brazil) in accordance with the manufacturer’s instructions. On postinfection day 5, the animals were anesthetized with ketamine (80 mg/kg body weight) and xylazine (5 mg/kg body weight), tracheotomized, paralyzed (vecuronium bromide, 0.005 mg/kg intravenously), and ventilated with a constant flow ventilator (Samay VR15; Universidad de la Republica, Montevideo, Uruguay) set to the following parameters: rate 100 breaths/minute, tidal volume (V_T_) 0.2 ml, and fraction of inspired oxygen (FiO_2_) 0.21. The anterior chest wall was surgically removed and a positive end-expiratory pressure of 2 cmH_2_O was applied. Airflow and tracheal pressure (Ptr) were measured. Lung mechanics were analyzed by the end-inflation occlusion method. In an open chest preparation, Ptr reflects transpulmonary pressure (P_L_). Static lung elastance (Est,L) was determined by dividing the elastic recoil pressure (Pel) by V_T_. Lung mechanics parameters were measured ten times in each animal. All data were analyzed using ANADAT software (RHT-InfoData, Inc., Montreal, QC, Canada). All experiments lasted less than 15 minutes. Blood samples were then collected via cardiac puncture into heparinized tubes and centrifuged at 600 × *g* for 5 minutes to separate plasma. Urine and blood samples were assayed to determine the levels of creatinine (Gold Analisa kit 427E; Gold Analisa Diagnóstica, Belo Horizonte, MG, Brazil) and blood urea nitrogen (BUN; Gold Analisa kit 335; Gold Analisa Diagnóstica, Belo Horizonte, MG, Brazil). The glomerular filtration rate (GFR) was derived from the creatinine clearance.

Prior to removal of the brain, liver, kidney, spleen, and lungs, a laparotomy was performed, heparin injected intravenously, the trachea clamped at end expiration, and the abdominal aorta and vena cava sectioned to kill the animals by exsanguination.

### Brain, spleen, liver, kidney, and lung histology

The brain, liver, kidney, spleen, and lungs were fixed in 4 % buffered formaldehyde, embedded in paraffin, and cut into 4-μm-thick slices, which were stained with hematoxylin and eosin (Vetec Química Fina, Rio de Janeiro, Brazil). A five-point, semiquantitative, severity-based scoring system was used to assess the degree of injury as follows: 0 = normal tissue; 1 = damage to 1–25 % of total tissue examined; 2 = damage to 26–50 % of total tissue examined; 3 = damage to 51–75 % of total tissue examined; and 4 = damage to 76–100 % of total tissue examined. The following parameters were analyzed: presence of malaria pigment, inflammation, fibrosis, and histoarchitectural damage. Lung histology was also examined using an integrating eyepiece with a coherent system consisting of a grid with 100 points and 50 lines of known length coupled to a conventional light microscope (Olympus BX51; Olympus Latin America Inc., São Paulo, SP, Brazil). The number of mononuclear and polymorphonuclear cells in pulmonary tissue was determined by the point-counting technique [17] across ten random, noncoincident microscopic fields [18]. These analyses were performed by an expert in lung pathology blinded to the experimental protocol.

### ELISA in lung tissue

Levels of tumor necrosis factor (TNF)-α, interferon (IFN)-γ, chemokine (C-X-C motif) ligand (CXCL)-1, hepatocyte growth factor (HGF), TGF-β, and vascular endothelial growth factor (VEGF) were quantified in lung tissue. Briefly, the lungs were excised and homogenized in cell lysis buffer (20 mM Tris, 150 mM NaCl, 5 mM KCl, 1 % Triton X-100, protease inhibitor cocktail (1:1000); Sigma-Aldrich), and immediately frozen at −80 °C. The total protein content of each tissue homogenate was evaluated by the Bradford method, followed by determination of cytokine production by a standard sandwich ELISA, performed in accordance with manufacturer instructions (R&D Systems, Minneapolis, MN, USA). Plates were read at 490 nm in an M5 Spectrophotometer (Molecular Devices, Sunnyvale, CA, USA).

### Statistical analysis

Survival analysis was performed using the Kaplan–Meier method and the log-rank (Mantel–Cox) test. Comparison between groups was performed using two-way analysis of variance followed by Tukey’s test. Parametric data were expressed as mean ± standard deviation (SD), and nonparametric data were expressed as median and interquartile range. All tests were performed using PASW Statistics for Windows, Version 18.0 (SPSS Inc., Chicago, IL, USA). Statistical significance was established as *p* <0.05.

## Results

### BM-MSC treatment increased survival rate in *P. berghei*-infected mice

**Fig. 1:**
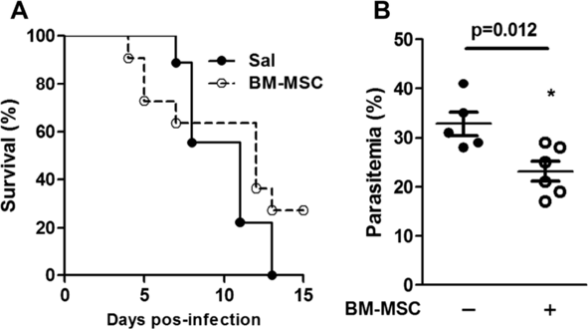
Survival rate and parasitemia of *P. berghei*-infected mice. Mice were infected with 5 × 10^6^ parasitized RBCs or mock-infected with saline and, 24 hours after infection, were treated with BM-MSCs. **a** Survival of mice infected with *P. berghei*. **b** Parasitemia was analyzed 5 days after infection using light microscopy. Values are expressed as means ± SD of six animals per group out of three experiments. *BM-MSC* bone marrow-derived mesenchymal stromal cell, *Sal* saline

All untreated *P. berghei*-infected mice succumb to infection within 12 days. Even though BM-MSC therapy appeared to increase the mortality rate at early time points, evaluation of the full time course allows for the conclusion that BM-MSC therapy increased survival as compared with nontreatment of *P. berghei*-infected mice (Fig. 1a). At day 5 after infection, parasitemia was analyzed, and treatment with BM-MSCs was found to have decreased parasitemia levels (Fig. 1b).

### Cerebral damage observed during ECM was not modulated by BM-MSC administration

**Fig. 2:**
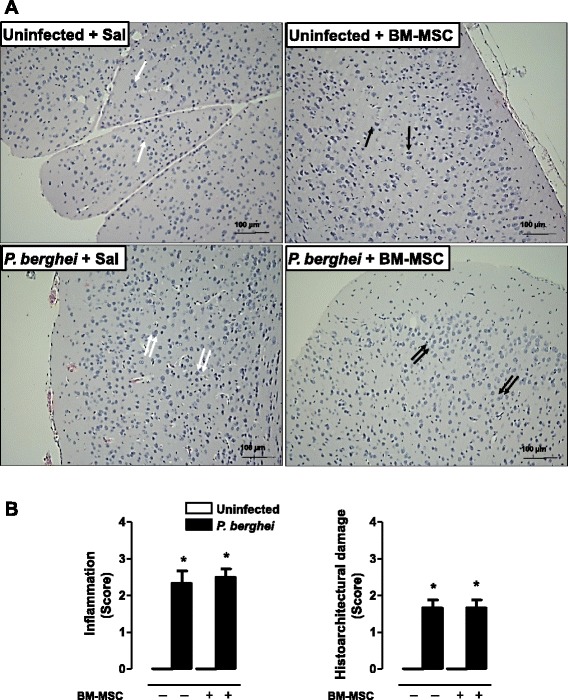
**a** Photomicrographs of brain tissue stained with hematoxylin and eosin. Original magnification × 1000; bars = 100 μm. Mice were inoculated with 5 × 10^6^ parasitized RBCs or saline and treated with BM-MSCs. Brains were excised 5 days after infection. Normal brain cortex with neurons, astrocytes, and oligodendrocytes (*single white arrows*). Treatment with BM-MSCs did not affect the brain cortex, which displays normal neurons, astrocytes, and oligodendrocytes (*black single arrows*). In *P. berghei*-infected mice treated with saline, neurons were damaged, with an increased number of astrocytes and oligodendrocytes (*double white arrows*). In *P. berghei*-infected mice treated with BM-MSCs, brain damage was repaired, with an increased number of astrocytes and oligodendrocytes within neutrophils (*double black arrows*). **b** A semiquantitative, severity-based score was used to measure inflammation and histoarchitectural damage in brains of mice infected with *P. berghei* or mock-infected with saline. Twenty-four hours after infection, mice were treated with BM-MSCs. Values are expressed as median (interquartile range) of six animals in each group. *Significantly different from uninfected group (*p* <0.05). *BM-MSC* bone marrow-derived mesenchymal stromal cell, *Sal* saline

Photomicrographs of brain tissue (Fig. 2a) from uninfected and *P. berghei-*infected mice treated or not with BM-MSC were obtained on postinfection day 5. Brains collected from both groups of uninfected mice (treated or not with BM-MSCs) exhibited similar histological patterns, characterized by normal brain cortex with neurons, astrocytes, and oligodendrocytes. In brains from *P. berghei*-infected mice we observed neuron damage associated with an increased number of astrocytes and oligodendrocytes. In brains from BM-MSC-treated *P. berghei*-infected mice there was a further increase in the number of astrocytes and oligodendrocytes, suggesting tissue repair. Semiquantitative analysis of brain damage revealed no significant differences in tissue inflammation or degree of histoarchitectural damage between the treated and nontreated *P. berghei*-infected groups (Fig. 2b). Neither malaria pigment nor fibrosis was detected in brain tissue of animals from either of the studied groups.

### BM-MSC treatment increased clearance of parasitized RBCs

**Fig. 3:**
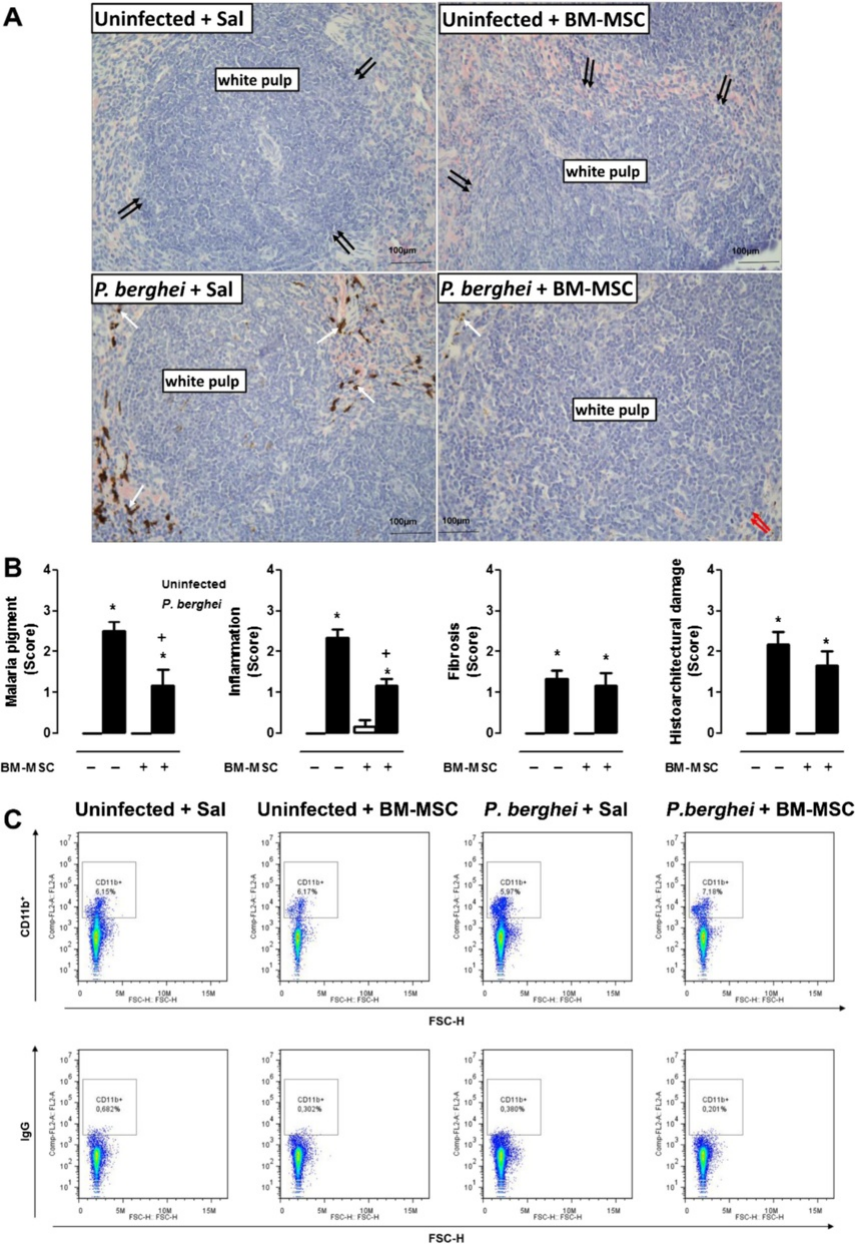
**a** Photomicrographs of spleen tissue stained with hematoxylin and eosin. Original magnification × 1000; bars = 100 μm. Mice were inoculated with 5 × 10^6^ parasitized RBCs or saline and treated with BM-MSCs. Spleens were excised 5 days after infection. Normal spleen architecture with white pulp (*double black arrows*). Uninfected mice treated with BM-MSCs also displayed normal spleen architecture (*double black arrows*). In *P. berghei-*infected mice, spleen damage was observed with activation of lymphocytes in white pulp, increased deposition of malaria pigment in red pulp (*single white arrows*), and increased number of lymphoblasts and lymphocytes in white pulp (*red double arrows*). **b** A semiquantitative, severity-based score was used to measure malaria pigment deposition, inflammation, fibrosis, and histoarchitectural damage in spleens of mice infected with *P. berghei* or mock-infected with saline and, 24 hours after infection, treated with BM-MSCs. Values are expressed as median (interquartile range) of six animals in each group. *Significantly different from uninfected group (*p* <0.05). ^+^Significantly different from *P. berghei*-infected group (*p* <0.05). **c** Representative dot-plots demonstrating CD11b^+^ fluorescence in splenocytes. *BM-MSC* bone marrow-derived mesenchymal stromal cell, *FSC* forward scatter, *Sal* saline

Spleens from *P. berghei*-infected mice showed evidence of tissue damage, with activation of lymphocytes in white pulp and increased deposition of malaria pigment in red pulp (Fig. 3b). BM-MSC administration did not affect spleen histology in uninfected mice (Fig. 3a, b); however, administration of BM-MSCs reduced levels of malaria pigment and increased the CD11b^+^ cell count in spleens of *P. berghei*-infected mice (Fig. 3c).

### BM-MSC treatment increased the number of Kupffer cells in liver

**Fig. 4:**
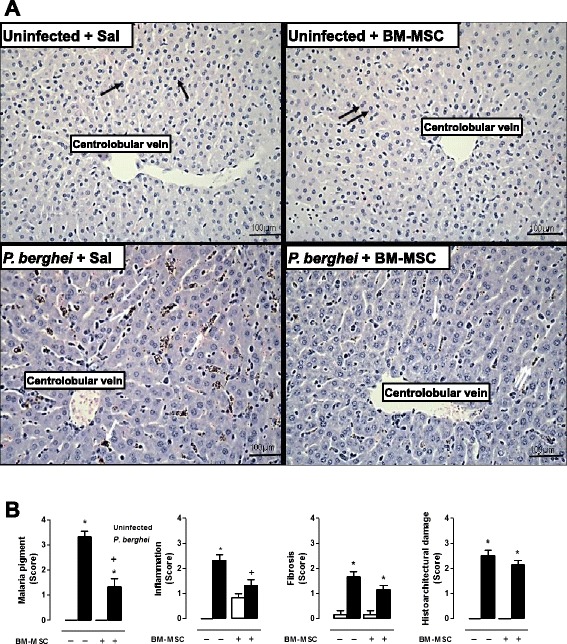
**a** Photomicrographs of liver tissue stained with hematoxylin and eosin. Original magnification × 1000; bars = 100 μm. Mice were inoculated with 5 × 10^6^ parasitized RBCs or saline and treated with BM-MSCs. Livers were excised 5 days after infection. Note the intact hepatocytes (*single black arrows*) involving the centrolobular vein. Administration of BM-MSCs did not alter the liver architecture (*double black arrows*) or centrolobular vein in control mice. *P. berghei*-infected, saline-treated mice exhibited hepatocyte derangement (*double white arrowhead*), increased deposition of malaria pigment, and an increased number of Kupffer cells (*single white arrows*). BM-MSC therapy increased the number of regenerated hepatocytes and Kupffer cells (*double white arrows*). **b** A semiquantitative, severity-based score was used to measure malaria pigment deposition, inflammation, fibrosis, and histoarchitectural damage in livers of mice infected with *P. berghei* or mock-infected with saline and, 24 hours after infection, treated with BM-MSCs. Values are expressed as median (interquartile range) of six animals in each group. *Significantly different from uninfected group (*p* <0.05). ^+^Significantly different from *P. berghei*-infected group (*p* <0.05). *BM-MSC* bone marrow-derived mesenchymal stromal cell, *Sal* saline

Administration of BM-MSCs to uninfected mice did not alter the liver architecture (Fig. 4a). In *P. berghei-*infected mice, we observed hepatocyte derangement, increased deposition of malaria pigment, and an increased number of Kupffer cells (Fig. 4b). After BM-MSC therapy, there was an increase in the number of regenerated hepatocytes and Kupffer cells (phagocytic cells) (Fig. 4a).

### BM-MSC treatment mitigated the histopathological features of *P. berghei*-induced kidney injury, but did not improve renal function

**Fig. 5:**
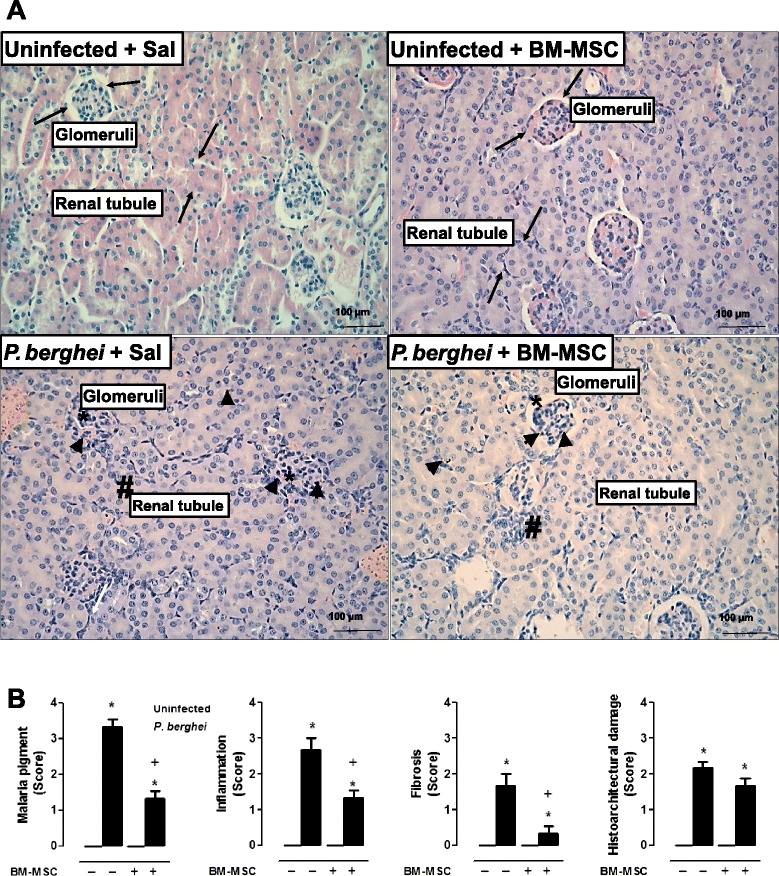
**a** Photomicrographs of kidney tissue stained with hematoxylin and eosin. Original magnification × 1000; bars = 100 μm. Mice were inoculated with 5 × 10^6^ parasitized RBCs or saline and treated with BM-MSCs. Uninfected mice treated or not with BM-MSCs also showed normal kidney architecture (*black arrows*). In *P. berghei*-infected mice treated with saline, mesangial proliferation occurred in the glomeruli (*) with hydropic degeneration of kidney tubular epithelium (#) and increased deposition of malaria pigment (*arrowhead*). In *P. berghei*-infected mice treated with BM-MSCs, normal mesangial cell architecture (*) and tubular cells (#), and sparse deposition of malaria pigment (*arrowhead*) were observed. **b** A semiquantitative, severity-based score was used to measure malaria pigment deposition, inflammation, fibrosis, and histoarchitectural damage in kidneys of mice infected with *P. berghei* or mock-infected with saline and, 24 hours after infection, treated with BM-MSCs. Values are expressed as median (interquartile range) of six animals in each group. *Significantly different from uninfected group (*p* <0.05). ^+^Significantly different from *P. berghei*-infected group (*p* <0.05). ^#^Significantly different from uninfected untreated group (*p* <0.05). *BM-MSC* bone marrow-derived mesenchymal stromal cell, *Sal* saline

Administration of BM-MSCs to uninfected mice did not affect the normal kidney architecture (Fig. 5a, b). *P. berghei* infection induced mesangial proliferation in the glomeruli accompanied by increased deposition of malaria pigment (Fig. 5a, b). *P. berghei*-infected mice treated with BM-MSCs exhibited restoration of mesangial cell architecture and tubular cells, as well as decreased deposition of malaria pigment (Fig. 5b).

**Fig. 6:**
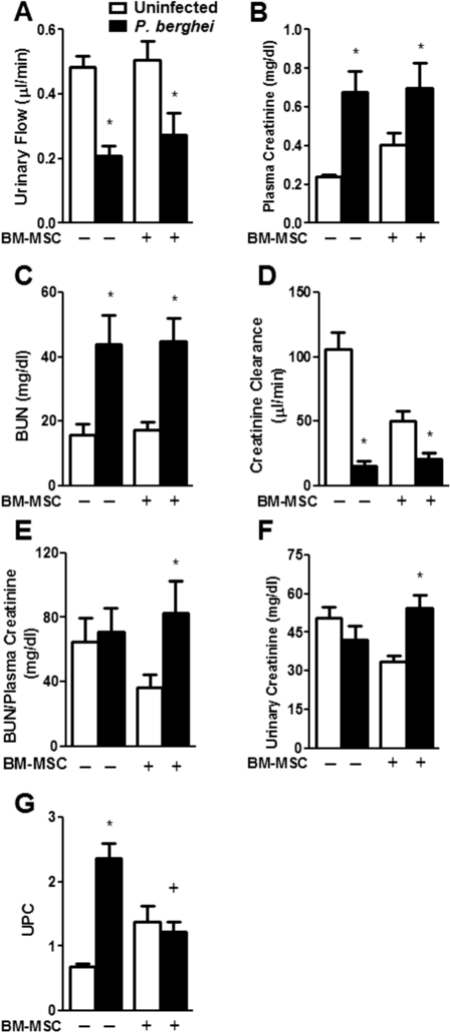
Effects of BM-MSC therapy on renal function in *P. berghei*-infected animals. Mice were infected with *P. berghei* or mock-infected with saline and, 24 hours after infection, treated with BM-MSCs. Five days after infection, plasma and urine samples were collected for analysis of renal function. Analysis of **a** urinary flow, **b** plasma creatinine, **c** blood urea nitrogen (BUN), **d** creatinine clearance, **e** BUN/plasma creatinine, **f** urinary creatinine, and **g** urinary protein/creatinine ratio (UPCr) in different experimental groups is depicted. Values are expressed as mean ± SD of six animals per group out of two experiments. *Significantly different from uninfected group (*p* <0.05). ^+^Significantly different from *P. berghei*-infected group (*p* <0.05). *BM-MSC* bone marrow-derived mesenchymal stromal cell, *UPC* urinary protein/creatinine

Concerning renal function, *P. berghei*-infected mice exhibited a 50 % reduction in urinary flow (Fig. 6a), while there was a threefold increase in serum creatinine (Fig. 6b) and BUN (Fig. 6c) as compared with the uninfected group. The alterations in urinary flow and serum creatinine were reflected by a sixfold decrease in creatinine clearance (Fig. 6d). No changes in the BUN/serum creatinine ratio or in urinary creatinine levels were observed in the infected group (Fig. 6e and f, respectively). BM-MSC treatment in infected mice did not change any of these parameters. Interestingly, uninfected mice that received BM-MSCs had increased serum creatinine and a significant reduction in urinary creatinine. The baseline creatinine clearance of uninfected mice was therefore reduced by BM-MSC treatment. The enhancement in serum creatinine levels observed in uninfected mice treated with BM-MSCs led to a decrease in the BUN/serum creatinine ratio. Furthermore, we observed that the increased urinary protein/creatinine ratio (UPCr) induced by *P. berghei* was significantly lower after BM-MSC treatment (Fig. 6g).

### BM-MSC improved lung mechanics and reduced lung inflammation in *P. berghei*-infected mice

**Fig. 7:**
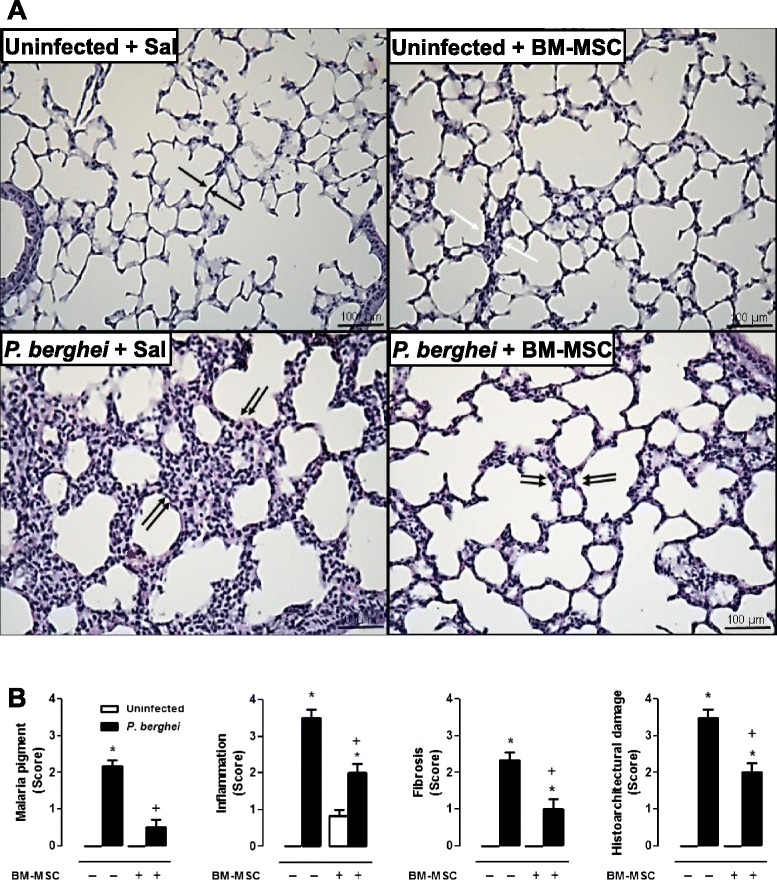
**a** Photomicrographs of lung parenchyma stained with hematoxylin and eosin. Original magnification × 1000; bars = 100 μm. In uninfected mice treated or not with BM-MSCs, normal architecture was observed with intact alveolar–capillary barrier (*black arrows* and *white arrows*, respectively). *P. berghei* infection induced lung damage, associated with structural disarrangement, thickening of the alveolar–capillary barrier by mononuclear cells (*double black arrows*) and malaria pigment deposition (*red arrowhead*), and increased number of fibroblasts (*double black arrows*). Treatment with BM-MSCs reduced thickening of the alveolar–capillary barrier and decreased the number of myofibroblasts within interstitial tissue (*double black arrow*) and collagen deposition (*double black arrows*). **b** A semiquantitative, severity-based score was used to measure malaria pigment deposition, inflammation, fibrosis, and histoarchitectural damage in lungs of mice infected with *P. berghei* or mock-infected with saline and, 24 hours after infection, treated with BM-MSCs. Values are expressed as the median (interquartile range) of six animals in each group. *Significantly different from uninfected group (*p* <0.05). ^+^Significantly different from *P. berghei*-infected group (*p* <0.05). *BM-MSC* bone marrow-derived mesenchymal stromal cell, *Sal* saline

**Fig. 8:**
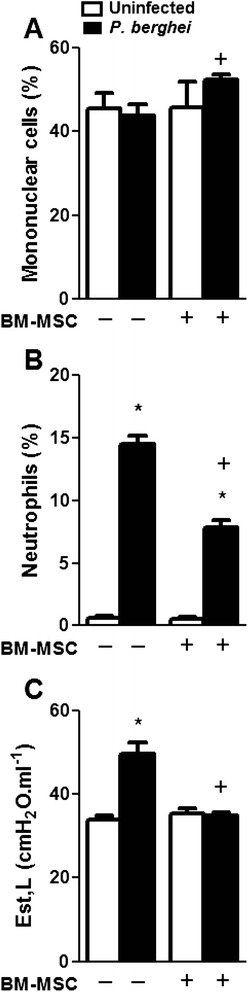
Fraction area of mononuclear cells (**a**) and neutrophils (**b**). Static lung elastance (Est,L) (**c**). Values are the mean ± SD of six animals in each group. *Significantly different from uninfected group (*p* <0.05). ^+^Significantly different from *P. berghei*-infected group (*p* <0.05). *BM-MSC* bone marrow-derived mesenchymal stromal cell

*P. berghei-*infected mice also exhibited lung damage, as characterized by structural derangements, thickening of the alveolar–capillary membrane, increased mononuclear cell and fibroblast counts, and malaria pigment deposition (Fig. 7a, b). After BM-MSC administration, reductions were observed in the thickness of the alveolar–capillary barrier, the number of myofibroblasts within interstitial tissue, collagen deposition (Fig. 7a), and neutrophil counts (Fig. 8b); however, there was an increase in the number of mononuclear cells (Fig. 8a).

Static lung elastance (Est,L) was increased in *P. berghei-*infected mice when compared with uninfected mice (Fig. 8c). BM-MSC administration reduced Est,L in *P. berghei*-infected mice.

**Fig. 9:**
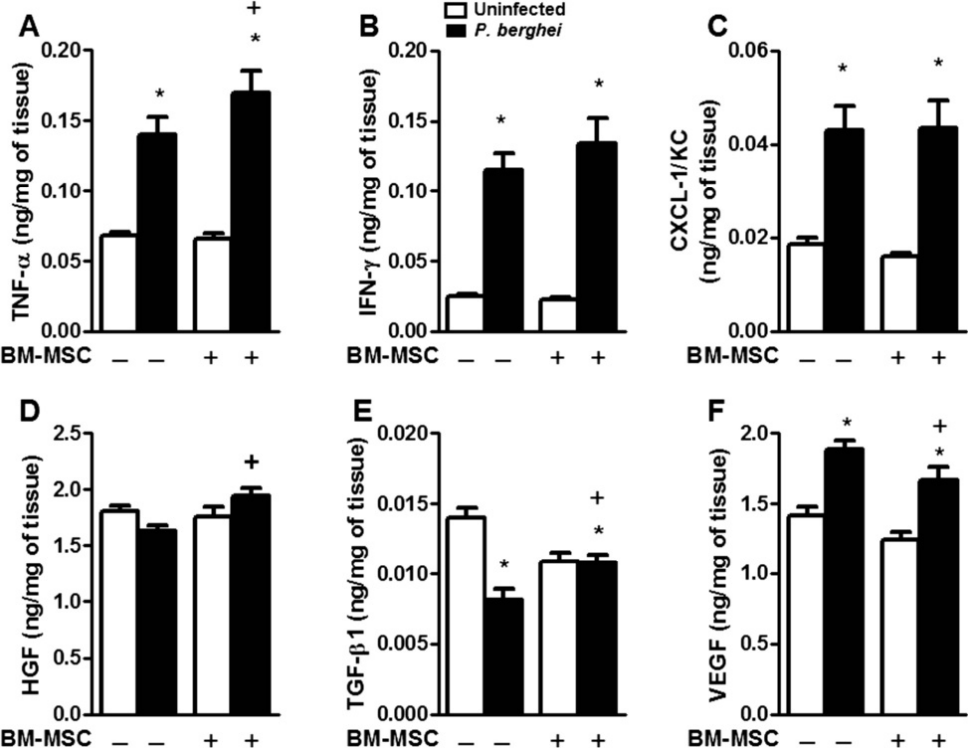
Cytokine production in lung tissue after BM-MSC treatment of *P. berghei-*infected mice. **a** Tumor necrosis factor (TNF)-α, **b** interferon (IFN)-γ, **c** chemokine C-X-C motif (CXCL)1/KC, **d** hepatocyte growth factor (HGF), **e** transforming growth factor (TGF)-β, and **f** vascular endothelial growth factor (VEGF) were evaluated by ELISA. Results are expressed as the mean ± SD of six animals per group. *Significantly different from uninfected group (*p* < 0.05). ^+^Significantly different from *P. berghei*-infected group (*p* < 0.05). *BM-MSC* bone marrow-derived mesenchymal stromal cell

*P. berghei*-infected mice exhibited increased levels of TNF-α, IFN-γ, CXCL-1/KC, and VEGF and reduced levels of TGF-β1 in lung tissue as compared with uninfected animals. No significant differences in HGF levels were observed between the *P. berghei*-infected and uninfected groups. BM-MSC administration increased TNF-α, TGF-β1, and HGF levels and reduced VEGF levels, but did not modulate the production of IFN-γ or CXCL-1/KC in *P. berghei*-infected mice (Fig. 9).

## Discussion

In the model of ECM employed in this study, the reduction in mortality rate observed after BM-MSC therapy was not associated with a reduction in brain damage. Even though histological evidence of lung, liver, kidney, and spleen injury was reduced, only lung function improved after therapy. These morphological and functional changes were not associated with a reduction in proinflammatory mediators, but rather with decreased peripheral parasitemia.

The use of BM-MSCs in diseases characterized by brain dysfunction has been extensively described. Authors have reported that BM-MSCs exert a beneficial effect in a paracrine fashion, by enhancing synaptic transmission and ameliorating microglial signaling [19–21]. In addition, the use of BM-MSCs as a therapeutic approach that targets host defenses has been proposed in bacterial diseases, including tuberculosis [22] and parasitic diseases [10, 11]. It is interesting to note that the parasitic conditions in which BM-MSC therapy has been studied are characterized by involvement of specific organs, such as the liver and heart, which reinforces the need for studies of parasitosis that feature multiple organ dysfunction, such as malaria. Indeed, previous studies concerning cell therapy during malaria infection have already been performed [12, 13, 23]. However, our study was the first designed to evaluate the effects of BM-MSC therapy in multiple organ dysfunction during ECM.

Accordingly, for the present study we used a model of ECM known to be associated with multiple organ dysfunction [24]. Mice succumb to ECM between 5 and 10 days post infection. Animals that survive longer than 7 days are considered resistant, and die from pathological conditions not related to ECM [4]. BM-MSCs were therefore administered 24 hours after *P. berghei* infection, when the inflammatory process in different organs was already present and intense. If treatment was started late in the course of malaria, when the mortality rate is high, this would have hampered interpretation of results. Mice were euthanized 5 days post infection because, at this time point, several pathological conditions were already present [25] but the mice were still alive; this approach prevented misinterpretation of results due to the presence of resistant animals.

Histologically, brains from mice with ECM exhibit cortical edema, congested capillaries, increased numbers of microglial cells, and glial cell swelling [26–28]. In addition, Nacer et al. [29] proposed recently that intracranial hypertension plays a crucial role in ECM development. The authors suggest that intracranial hypertension could be promoted by the presence of late-stage infected erythrocytes, i.e.*,* schizonts, in postcapillary venules. Interestingly, the reduction in parasitemia shown in Fig. 1 was mainly due to reduction of early-stage (i.e.*,* ring) forms, but not schizonts, which may explain why BM-MSC treatment did not improve brain tissue damage despite increasing the survival rate in *P. berghei*-infected mice. On the other hand, the number of astrocytes and oligodendrocytes was further increased, which suggests tissue repair [30]. Glial cells have been described as undergoing apoptosis during ECM [31]; nevertheless, the outcome of ECM does not depend on the attenuation of glial cell dysfunction [32, 33], suggesting that this process is not involved in ECM development. Furthermore, glial cells increase neutrophil survival and phagocytosis, which could provide protection against brain infection [34]. Since cell-based therapy decreases parasite load in noncerebral malaria models [12, 13], we hypothesized that BM-MSC administration would stimulate phagocytosis and promote parasite clearance, which has been extensively described in the literature as occurring in the spleen [35, 36]. The spleen is well characterized as a hematopoietic site during experimental malaria [37]; the increased numbers of constitutive hematopoietic stem progenitor cells observed in the spleen during *P. berghei* infection impair parasitemia exacerbation and increase mouse survival [23]. This fact corroborates the hypothesis that treatment with BM-MSCs promotes parasite clearance and increases survival of infected mice. In the present study, we observed a reduction in the levels of malaria pigment in the spleen after BM-MSC therapy, providing further evidence for the aforementioned improvement in parasite clearance.

In our experiment, C57Bl/6 mice infected with *P. berghei* developed not only ECM but also kidney injury, which was characterized by reduced urinary flow and creatinine clearance [38]. This renal function impairment has been associated with increased parasitemia [39]. Interestingly, even though tissue parasitemia and inflammatory infiltration were reduced, BM-MSCs did not improve renal function. This dissociation between reduction in kidney damage and absence of improvement in renal function may be attributable to the duration of the analysis period, since, in previous studies, mice that survived ECM continued to exhibit evidence of kidney injury 21 days after clearance of parasitemia [38].

Some studies have reported that BM-MSCs attenuate lung inflammation and fibrosis as well as improve pulmonary function in noninfectious models [40–42]. A previous study concerning BM-MSC treatment of infectious diseases suggests that lung injury attenuation was associated with disease recovery [43]. Even though cell-based therapies have already been used in malaria infection, no study had thus far investigated the effects of BM-MSCs on ECM-associated lung damage. BM-MSCs reduced lung tissue parasitemia as well as neutrophil infiltration despite an increase in mononuclear cell counts. Additionally, BM-MSC treatment reduced lung tissue damage and fibrosis, thus improving pulmonary function, which may suggest an association between attenuation of general lung dysfunction attenuation and the outcome of ECM. In this context, it is known that any therapy which regulates the lung injury-induced inflammatory cascade may also reduce distal organ dysfunction [44]. The reduction of malaria pigment deposition in lung tissue could also result in decreased neutrophil accumulation, thus improving lung morphofunction. However, attenuation of lung injury after BM-MSC therapy is not exclusively associated with diminished parasitemia, since malaria-induced lung injury is not necessarily a direct consequence of parasitemia [45]. We thus speculate that BM-MSCs may improve lung morphofunction for several reasons: (1) intravenously administered BM-MSCs accumulate mainly in lung tissue [46]—more beneficial effects would thus occur in the lung than in other organs; (2) the decrease in tissue parasitemia would be associated with increased numbers of lung tissue phagocytic cells [47]; and (3) VEGF, which is the main factor implicated in malaria-induced lung injury [45], would be reduced. Corroborating these hypotheses, we observed that the number of macrophages was indeed increased and levels of VEGF were indeed reduced in lung tissue.

This study has some limitations that should be addressed. First, ECM was induced by *P. berghei* inoculation; thus, our findings cannot be extrapolated to other models of malaria associated with different degrees of severity or to human malaria. Second, BM-MSCs were administered 1 day after infection, when the severity of ECM was reduced compared with day 5 [25]. However, if treatment had been started late in the course of malaria, the mortality rate would be high, hampering interpretation of the results. Additionally, as noted above, mice that survive longer than 7 days are considered resistant and die of pathological conditions not related to cerebral malaria [4]. Third, the observation time was relatively short (5 days post infection), precluding evaluation of the dynamics of malaria-induced multiple organ dysfunction. However, several organs were already damaged at day 5—including the brain, as demonstrated by impaired behavior and cognition. Fourth, no specific antimalarial therapy was given with BM-MSCs because this study was designed as a proof of concept rather than an evaluation of optimal therapy. Fifth, BM-MSCs were not tracked within the organs because markers used for this purpose are usually present for up to 24 hours and our analysis was carried out on day 5, when many organs had already been affected by *P. berghei* infection.

## Conclusions

BM-MSC treatment increased survival and reduced parasitemia and malaria pigment deposition in the spleen, liver, kidney, and lung, but not in the brain. The two main organs associated with worse prognosis in malaria—the lung and the kidney—sustained less histological damage after BM-MSC therapy, with a more pronounced improvement in lung function.

## Abbreviations

ARDS: Acute respiratory distress syndrome
BBB: Blood–brain barrier
BM-MSC: Bone marrow mesenchymal stromal cell
BUN: Blood urea nitrogen
CXCL: Chemokine (C-X-C motif) ligand
DMEM: Dulbecco’s modified Eagle’s medium
ECM: Experimental cerebral malaria
ELISA: Enzyme-linked immunosorbent assay
Est,L: Static lung elastance
FBS: Fetal bovine serum
FiO_2_: Fraction of inspired oxygen
FITC: Fluorescein isothiocyanate
FSC: Forward scatter
GFR: Glomerular filtration rate
HGF: Hepatocyte growth factor
IFN: Interferon
i.p.: Intraperitoneally
mAb: Monoclonal antibody
MSC: Mesenchymal stromal cell
PBS: Phosphate-buffered saline
Pel: Elastic recoil pressure
P_L_: Transpulmonary pressure
Ptr: Tracheal pressure
RBC: Red blood cell
SD: Standard deviation
SSC: Side scatter
TGF: transforming growth factor
TNF: Tumor necrosis factor
UPCr: Urinary protein/creatinine ratio
VEGF: Vascular endothelial growth factor
V_T_: Tidal volume
